# *Cryptosporidium* animal species in Iran: a systematic review and meta-analysis

**DOI:** 10.1186/s41182-020-00278-9

**Published:** 2020-12-05

**Authors:** Mousa Motavalli Haghi, Zohreh Khorshidvand, Salman Khazaei, Faezeh Foroughi-Parvar, Hossein Sarmadian, Nastaran Barati, Fariborz Etemadifar, Reza Ghasemikhah

**Affiliations:** 1 Department of Medical Parasitology and Mycology, School of Medicine, Hamadan University of Medical Sciences, Hamadan, Iran; 2 Research Center for Health Sciences, Hamadan University of Medical Sciences, Hamadan, Iran; 3 Department of Infectious Disease, School of Medicine, Arak University of Medical Sciences, Arak, Iran; 4 Research and Technology, Hamadan University of Medical Sciences, Hamadan, Iran; 5 Department of Parasitology and Mycology, School of Medicine, Arak University of Medical Sciences, Arak, Iran

**Keywords:** *Cryptosporidium*, Intestinal parasites, Animal, Systematic review, Iran

## Abstract

**Background:**

Cryptosporidiosis is an acute and short-term infection which can lead to severe diarrhea (intestinal cryptosporidiosis) associated with a persistent cough in the host with immune system defect. This systematic review and meta-analysis was conducted to estimate the prevalence of animal *Cryptosporidium* species and the corresponding epidemiological aspects in Iran.

**Methods:**

In this study, all original research articles relating to the animal cryptosporidiosis in Iran were collected from reliable databases using keywords. A meta-analysis was conducted separately for each subgroup, and heterogeneity among the studies was performed using the *Q* and *I*^2^ tests. Furthermore, it should be noticed that the significance level in the statistical analysis with the Comprehensive Meta-analysis software was considered to be less than 0.05. Finally, meta-analysis results were shown in forest plot with a 95% CI.

**Results:**

In total, 4795 studies were included in the initial screening. Duplicated or non-original studies and the ones which did not meet our considered criteria were excluded from the list. Out of the 100 articles included in our first list for the meta-analysis, 40, 16, 13, 10, 9, 7, and 5 were done on cattle and calves, birds, dogs, sheep, rodents, camels, and horses, respectively. The prevalence rate of cryptosporidiosis among the birds, horses, rodents, camels, dogs, cattle, and sheep in Iran was estimated to be 7.5%, 19.5%, 20.8%, 8.4%, 4.9%, 14.4%, and 9.1%, respectively.

**Conclusion:**

The different *Cryptosporidium* species have been found in different regions of Iran. Geographical region, climate, and domestic animals are considered as factors responsible for animal cryptosporidiosis prevalence in the area. Moreover, this parasite is zoonotic which causes disease in animals as well as humans which can result in economic loss.

**Supplementary Information:**

The online version contains supplementary material available at 10.1186/s41182-020-00278-9.

## Introduction

Intestinal parasites are considered an important public health problem in humans and animals in developing and low-income countries [1–3]. *Cryptosporidium* is one of the most common intestinal protozoan parasites which is located in the phylum of *Apicomplexa* and causes cryptosporidiosis [2]. *Cryptosporidium* is spread easily in the environment due to its simple transmission via contaminated water, air, and dust. Cryptosporidiosis is considered as a major economic problem in many countries including Iran, and there are annually numerous reports of this infection in immunocompromised and young children [2–5]. Different methods have been used to detect the protozoan parasite including the molecular diagnostic method which is considered one of the most useful diagnostic tools.

This method has identified up to 30 species and more than 50 genotypes of *Cryptosporidium* [6–8]. Different species of *Cryptosporidium* have been reported in various hosts including birds, horses, cattle, sheep, camels, rodents, and dogs. *Cryptosporidium parvum*, *C. hominis*, *C. canis*, *C. felis*, *C. meleagridis*, and *C. muris* were distinguished from gastrointestinal diseases as well as diarrhea in humans [9]. However, the infection typically occurs in a short-term and acute form in immunocompromised and HIV-positive individuals. In these cases, it tends to remain in the lower intestine for up to 6 weeks with severe diarrhea and persistent cough [9]. This protozoan could develop its life cycle in one host without the requirement of other animals as intermediate or reservoir hosts [9, 10]. Despite the many studies which were conducted in Iran on investigating the prevalence of cryptosporidiosis in different animal hosts, these data have not shown the overall prevalence of animals in Iran. Since cryptosporidiosis causes irreversible economic damages to domestic animals, critical screening programs and epidemiological aspects should be considered by authorities. This systematic review and meta-analysis was conducted to estimate the prevalence of animal *Cryptosporidium* species and their epidemiological aspects in Iran.

## Materials and methods

### Study protocol

The present systematic review focused on the estimates of the prevalence of animal *Cryptosporidium* species according to the PRISMA guidelines for systematic review and meta-analysis [11] (Supplementary 1).

### Search strategy

In order to select the suitable articles for this study, all records since 1991 up to February 2018 were investigated using seven international databases in English including PubMed, Web of Science, Scopus, Science Direct, and Google Scholar search engine as well as national databases in Persian including Magiran (http://www.magiran.com/) and Scientific Information Database (SID) (http://www.sid.ir/). Furthermore, references of each article were screened manually, and the authors were contacted for additional references.

The databases screening was performed using the following keywords: prevalence, *Cryptosporidium*, cryptosporidiosis, animal *Cryptosporidium*, *Cryptosporidium* species, *C. parvum*, *C.hominis*, animal, cattle, calf, sheep, goat, camel, horse, rodent, bird, chicken, epidemiology, Iran, serology, PCR, and molecular (Box 1).

**Box 1:** Search strategy for MEDLINE (Mesh, Medical Subject Headings)

1: Prevalence [Text Word] OR Prevalence [Mesh Term]	
2: Epidemiology [Text Word] OR Epidemiology [Mesh Term]	
2: Cryptosporidium [Text Word] OR Cryptosporidium [Mesh Terms]	
3: Cryptosporidiosis [Text Word] OR Cryptosporidiosis [Mesh Term]	
4: Animal [Text Word] OR Animal [Mesh Term]	
5: Cattle or Calf or Sheep or Goat or Camel or Horse or Rodent or Bird or Chicken	
6: Iran	
7: Serology [Text Word] OR Serology [Mesh Term]	
8: PCR [Text Word] OR PCR [Mesh Term]	
7: Microscopic [Text Word] OR Microscopic [Mesh Term]	
5: Meta-analysis [Text Word] OR Meta-analysis [Mesh Terms]	
6: 1 AND 2 AND 3 AND 4 AND 5 AND 6 AND 7	

### Eligibility criteria

All original descriptive studies which investigated the prevalence of *Cryptosporidium* in animals in Iran, both in English and Persian, were included in this study. Duplicates, qualitative studies, review articles, case reports, case series, and studies out of Iran or those performed on humans were excluded. Finally, articles with epidemiological parameters of interest were selected, and a total of 100 collected articles fulfilled the considered criteria.

### Quality assessment

The scoring system based on the 8-item modified Newcastle Ottawa Scale (NOS) for non-randomized studies was used for assessing the quality of the studies. In this system, each question has a score between 0 and 1, and the maximum point summation is 9. Studies with point summation 5 or less, 6–7, and 8–9 were considered low, moderate, and high quality, respectively [12].

### Screening and data extraction

All records were evaluated based on their title and abstract and according to the inclusion and exclusion criteria by two researchers (MM and NB) independently. The kappa index showed an agreement of 91% between the findings of two researchers. The full-text version of the papers was obtained through library resources and online databases. Finally, the difference between records among the researchers was corrected by re-examining the articles. The agreement was reached by group discussion with a third researcher (SK).

Data extraction was conducted independently by two researchers (MM and NB) and imported to the pre-prepared form. Data including authors, year of study, publication year, kind of animal, geographical area of the study, number of examined, number of positive, prevalence rate, and type of host were extracted from articles.

### Quality assessment studies

The methodological quality of the studies was examined based on the guidelines of the Newcastle and Ottawa Statements [12]. This guideline sets the criteria for selecting people to study, comparing and accepting them, as well as exposure and consequences where a maximum of 9 stars can be allocated to each study. Studies with 7 stars or more are classified as high-quality studies, and studies with 6 stars and less are considered as low-quality studies. Investigating the probability of an error in the results of the studies is performed separately by two researchers. The disagreement between the parties is resolved through negotiations.

### Data analysis

The meta-analysis method was adopted to a 95% confidence interval (CI) in order to assess the pooled prevalence of *Cryptosporidium* infection in animals using the random effect model. Various subgroup analyses were separately conducted based on animal type and the associated species. Finally, meta-analysis results were displayed in forest plot (reported as effect estimates (ES) with a 95% CI). We also performed a sensitivity analysis to verify the stability of the data. In order to assess the sensitivity analysis, the effect estimate was estimated irrespective of one study at a time, and the robustness of the pooled estimate was assessed. Heterogeneity was calculated among the studies by the *Q* and *I*^2^ tests [6, 8]. Cochran’s *Q* test (*Q* statistic, *p* < 0.10) showed statistically significant heterogeneity, and *I*^2^ statistic (*I*^2^ > 50%) indicates a large heterogeneity. Statistical analysis and data analysis were performed using the second version of the Comprehensive Meta-analysis software. The significance level was considered to be less than 0.05.

## Results

### Description of studies

**Fig. 1:**
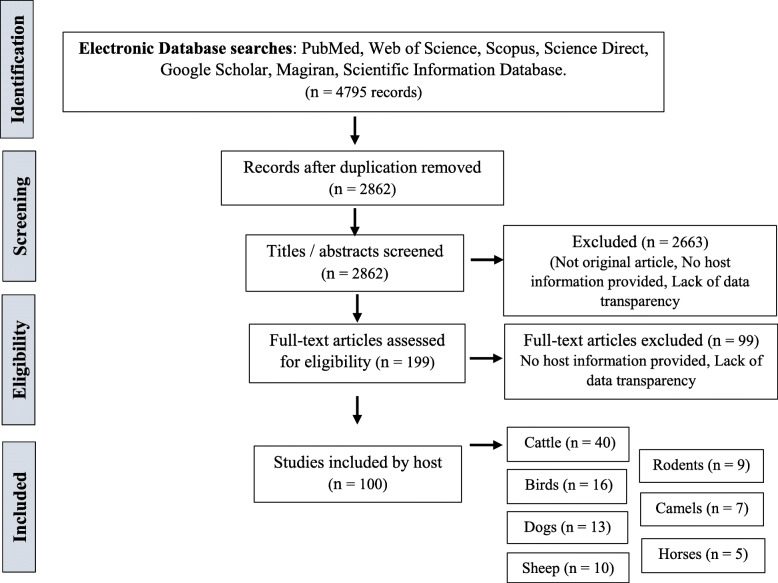
Flowchart describing the study design process

In total, 4795 studies were collected in the initial screenings from the published articles and their references in the screened databases up to February 2018. Specifically, 62, 124, 174, 62, 543, 10, and 3820 studies collected from PubMed, Web of Science, Scopus, Scientific Information Database, Magiran, Science Direct, and Google Scholar, respectively. A total of 1933 duplicated records and 5 studies which were not original articles (i.e., letter, commentary, review) were screened out. Regarding the relevance of the title and abstract to the purpose of the study, 2857 irrelevant studies were excluded. Accordingly, 2757 studies were retrieved for further assessment. Altogether, 100 articles were selected for the meta-analysis study. The display process and literature search results were presented respectively in Fig. 1 and Table 1.

**Table 1:** Baseline characteristics of included studies

First author	Publication year	Province	Animal	Samples	Positive samples	Prevalence (%)	Species	Laboratory method
Nouri [13]	1991	Lorestan	Sheep	276	37	13.4	*Cryptosporidium* spp.	Microscopic
Rezaeian [14]	1993	Tehran	Sheep	510	26	5.1	*Cryptosporidium* spp.	Microscopic
Fasihi Harandi [15]	2006	Kerman	Sheep	434	60	13.8	*Cryptosporidium* spp.	Microscopic
Vahedi [16]	2008	Mazandaran	Lamb	708	29	4.1	*Cryptosporidium* spp.	Microscopic
Heidari [17]	2012	Hamadan	Sheep	220	19	8.6	*Cryptosporidium* spp.	Microscopic
Khezri [18]	2013	Kurdistan	Lamb	850	87	10.2	*Cryptosporidium* spp.	Microscopic
Gharekhani [19]	2013	Hamedan Esfahan, Yazd Fars, Bushehr Mazandaran	Sheep	1749	198	11.3	*Cryptosporidium* spp.	Microscopic
Shafieyan [20]	2014	Lorestan	Sheep	345	20	5.8	*Cryptosporidium* spp.	Microscopic
Sadeghi [21]	2015	Kurdistan	Sheep	180	11	6.1	*Cryptosporidium* spp.	Microscopic
Dalimi [6]	2017	Tehran	Sheep	1300	22	1.7	*C. andersoni*, *C. parvum*	Nested-PCR
Radfar [22]	2006	Kerman	Calf	291	63	21.6	*Cryptosporidium* spp.	Microscopic
Maleki [23]	2006	Lorestan	Cattle	400	70	17.5	*Cryptosporidium* spp.	Microscopic
Parsa [24]	2007	Azerbaijan	Cattle	300	16	5.3	*C. andersoni*	Microscopic
Azami [25]	2007	Isfahan	Cattle	480	30	6.3	*Cryptosporidium* spp.	Microscopic
Azizi [26]	2007	Chaharmahl	Calf	400	72	18	*Cryptosporidium* spp.	Microscopic
Yakhchali [27]	2007	Kurdistan	Cattle	260	103	39.6	*Cryptosporidium* spp.	Microscopic
Keshavarz [28]	2008	Qazvin	Cattle	272	51	18.8	*C. andersoni*, *C. parvum*, *C. bovis*	PCR-RFLP
Mohamadi [29]	2008	Ardabil	Cattle	107	14	13.1	*C. andersoni*	Nested PCR&RFLP
		Ardabil	Cattle	107	5	4.7	*C. parvum*	Nested PCR&RFLP
Hassanpour [30]	2008	Azerbaijan	Calf	482	31	6.4	*Cryptosporidium* spp.	Microscopic
Fotouhi [31]	2008	Kerman	Cattle	412	78	18.9	*C. andersoni*, *C. parvum*	Microscopic
Vahedi [16]	2008	Mazandaran	Calf	713	28	3.9	*Cryptosporidium.* spp.	Microscopic
Shayan [32]	2008	Tehran	Cattle	173	64	37	*Cryptosporidium* spp.	Microscopic
Davoudi [33]	2009	Azerbaijan	Calf	50	7	14	*Cryptosporidium* spp.	Microscopic
			Rat		40	80		
Pirestani [34]	2009	Tehran	Calf	573	69	12	*Cryptosporidium* spp.	Microscopic
Ranjbar [35]	2009	Mazandaran	Calf	150	11	7.3	*Cryptosporidium* spp.	Microscopic
Fallah [36]	2009	Kurdistan	Calf	412	35	8.5	*Cryptosporidium* spp.	Microscopic
Baghban [37]	2009	Kohkiluyeh	Calf	80	37	46.3	*Cryptosporidium* spp.	Microscopic
Nourmohamadzadeh [38]	2010	Azerbaijan	Calf	500	207	41.4	*Cryptosporidium* spp.	Microscopic
Safavi Afshari [39]	2010	Khorasan	Calf	112	82	73.2	*Cryptosporidium* spp.	Microscopic
Ranjbar [40]	2011	Tehran	Calf	200	35	17.5	*Cryptosporidium* spp.	Microscopic
Changizi [41]	2011	Semnan	Cattle	200	21	10.5	*C. ryanae*	Microscopic
Bairami [42]	2011	Tehran	Cattle	100	30	30	*Cryptosporidium* spp.	PCR
Heidarnegadi [43]	2011	Khuzestan	Cattle	45	29	64.4	*Cryptosporidium* spp.	Microscopic
Ranjbar [44]	2013	Khorasan	Calf	400	10	5.2	*Cryptosporidium* spp.	Microscopic
Ghadrdan [45]	2011	Semnan	Calf	50	8	16	*Cryptosporidium* spp.	Microscopic
Heidari [46]	2012	Hamadan	Cattle	477	76	15.9	*Cryptosporidium* spp.	Microscopic
Ranjbar [47]	2012	Khorasan	Calf	170	19	11.2	*Cryptosporidium* spp.	Microscopic
Jafari [48]	2012	Hamedan	Calf	195	25	12.8	*Cryptosporidium* spp.	Microscopic
Asadpour [49]	2013	Khorasan	Calf	300	45	15	*C. parvum*	PCR-RFLP
Mirzai [50]	2013	Azerbaijan	Cattle	246	55	22.4	*C. andersoni*, *C. parvum*	Microscopic
Dalimi [51]	2013	Tehran	Cattle	940	23	2.4	*C. andersoni*	Nested PCR
Shafieyan [20]	2014	Lorestan	Cattle	430	39	9.1	*Cryptosporidium* spp.	Microscopic
Mojarad [52]	2014	Qazvin	Cattle	158	26	16.5	*Cryptosporidium* spp.	Microscopic
Bahrami [53]	2014	Khuzestan	Calf	90	41	45.6	*Cryptosporidium* spp.	Microscopic
Mahami oskouei [54]	2014	Ilam	Cattle	217	8	3.7	*C. parvum*	Nested PCR&RFLP
Mirzaghavami [55]	2015	Tehran	Cattle	50	12	24	*Cryptosporidium* spp.	Microscopic
Saki [1]	2017	Khuzestan	Cattle	240	5	2.1	*C. parvum*	Nested PCR&RFLP
Mosallanejad [56]	2010	Khuzestan	Dog	93	4	4.3	*C. parvum*	ELISA
Kake khani [57]	2011	Ilam	Dog	112	8	7.1	*Cryptosporidium* spp.	Microscopic
Heidari [17]	2012	Hamadan	Dog	210	8	3.8	*Cryptosporidium* spp.	Microscopic
Badrooj [58]	2012	Shiraz	Dog	29	0	1.7	*Cryptosporidium* spp.	PCR
Beiromvand [59]	2012	Khorasan	Dog	77	4	5.2	*Cryptosporidium* spp.	Microscopic
Mirzaei [60]	2013	Kerman	Dog	548	11	2	*Cryptosporidium* spp.	Microscopic
				100	3	3	*Cryptosporidium* spp.	Microscopic
Gharekhani [61]	2014	Hamedan	Dog	210	8	3.8	*Cryptosporidium* spp.	Microscopic
Arzamani [62]	2016	Khorasan	Dog	32	1	3.1	*Cryptosporidium* spp.	Microscopic
Tavalla [63]	2017	Khuzestan	Dog	350	43	12.3	*Cryptosporidium* spp.	PCR
Mohaghegh [64]	2017	Kermanshah	Dog	301	72	23.9	*Cryptosporidium* spp.	Microscopic
Ranjbar [9]	2018	Isfahan	Dog	140	3	2.1	*C. parvum*	PCR
Borji [65]	2009	Khorasan	Camel	306	6	2	*Cryptosporidium* spp.	Microscopic
Behzadi [66]	2009	Isfahan	Camel	103	39	37.9	*Cryptosporidium* spp.	Microscopic
Nazifi [67]	2009	Hormozgan	Camel	65	11	16.9	*Cryptosporidium* spp.	Microscopic
Sazmand [68]	2011	Yazd	Camel	300	61	20.3	*Cryptosporidium* spp.	Microscopic
Yakhchali [69]	2012	Azerbaijan	Camel	170	17	10	*Cryptosporidium* spp.	Microscopic
Radfar [70]	2012	Kerman	Camel	85	2	2.4	*C. parvum*	ELISA
Shahraki [71]	2015	Sistan	Camel	184	1	0.5	*C. parvum*	ELISA
Hamedi [72]	2003	Hormozgan	Rat	63	11	17.5	*Cryptosporidium* spp.	Microscopic
Shiraz i[73]	2009	Azerbaijan	Rat	50	37	74	*Cryptosporidium* spp.	Microscopic
Davoudi [32]	2010	Azerbaijan	Rat	50	40	80	*Cryptosporidium* spp.	Microscopic
Bahrami [74]	2012	Tehran	Rat	77	21	27.3	*C. parvum*	PCR-RFLP
Borji [75]	2013	Khorasan	Hamster	100	44	44	*Cryptosporidium* spp.	Microscopic
Mirzaghavami [56]	2015	Tehran	Rat	180	23	12.8	*Cryptosporidium* spp.	Microscopic
Saki [1]	2016	Khuzestan	Rodent	100	3	3	*C. parvum*	Nested PCR&RFLP
Valipour [76]	2016	Khuzestan	Rat	42	3	7.1	*Cryptosporidium* spp.	Microscopic
Mohebali [77]	2017	Azerbaijan	Rodent	204	1	0.5	*Cryptosporidium* spp.	Microscopic
Banani [78]	2000	Fars	Chicken	1522	125	8.2	*C. bailey*	ELISA
Mirzai [79]	2008	Kerman	Pigeon	400	10	2.5	*Cryptosporidium* spp.	Microscopic
Behzadi [67]	2009	Isfahan	Ostrich	75	21	28	*Cryptosporidium* spp.	Microscopic
Norolahi Fard [80]	2010	Khorasan	Pigeon	200	5	2.5	*Cryptosporidium* spp.	Microscopic
Shemshadi [81]	2010	Semnan	Broiler	240	57	23.8	*Cryptosporidium* spp.	Microscopic
Haghbin [82]	2010	Mazandaran	Broiler	300	39	13	*Cryptosporidium* spp.	Microscopic
Radfar [83]	2011	Khorasan	Pigeon	102	3	2.9	*Cryptosporidium* spp.	Microscopic
Heidarnegadi [42]	2011	Khuzestan	Turkey	22	11	50	*Cryptosporidium* spp.	Microscopic
Heidari [17]	2012	Hamadan	Poultry	200	5	2.5	*Cryptosporidium* spp.	Microscopic
Hamidinejat [84]	2014	Lorestan	Chicken	1000	7	0.7	*C. bailey*	PCR-RFLP
Hashemzade [85]	2014	Azerbaijan	Bird	400	36	9	*Cryptosporidium* spp.	Microscopic
Hashemzade [86]	2014	Azerbaijan	Poultry	400	21	5.3	*Cryptosporidium* spp.	Microscopic
Mirzaghavami [56]	2015	Tehran	Pigeon	40	1	2.5	*Cryptosporidium* spp.	Microscopic
Shemshadi [87]	2016	Rasht	Duck	30	5	16.7	*C. bailey*	Microscopic
Soltanialvar [88]	2016	Khuzestan	Turkey	200	4	2	*Cryptosporidium* spp.	Microscopic
Larki [89]	2017	Khuzestan	Duck	41	11	26.8	*Cryptosporidium* spp.	Microscopic
Naghibi [90]	2002	Khorasan	Horse	300	80	26.7	*Cryptosporidium* spp.	Microscopic
Tavassoli [91]	2005	Azerbaijan	Horse	221	35	15.8	*Cryptosporidium* spp.	Microscopic
Mirian [5]	2010	Tehran	Horse	200	50	25	*Cryptosporidium* spp.	Microscopic
Heidari [17]	2012	Hamedan	Horse	158	20	12.7	*Cryptosporidium* spp.	Microscopic
Ghadrdan [92]	2012	Khuzestan	Horse	100	18	18	*Cryptosporidium* spp.	Microscopic

Within these 100 articles, 40, 16, 13, 10, 9, 7, and 5 studies were performed on cattle, birds, dogs, sheep, rodents, camels, and horses, respectively. The most frequent studies were performed on cattle and the least ones on horses. Considering the various projects in searching cryptosporidiosis on cattle in Iran, the distribution of positive cases relating to cattle is presented in Fig. 9. The quality assessment of studies using the guideline of the Newcastle Ottawa Scale showed that 27%, 65%, and 8% of the studies have low, medium, and high quality, respectively.

### Main analysis

**Fig. 2:**
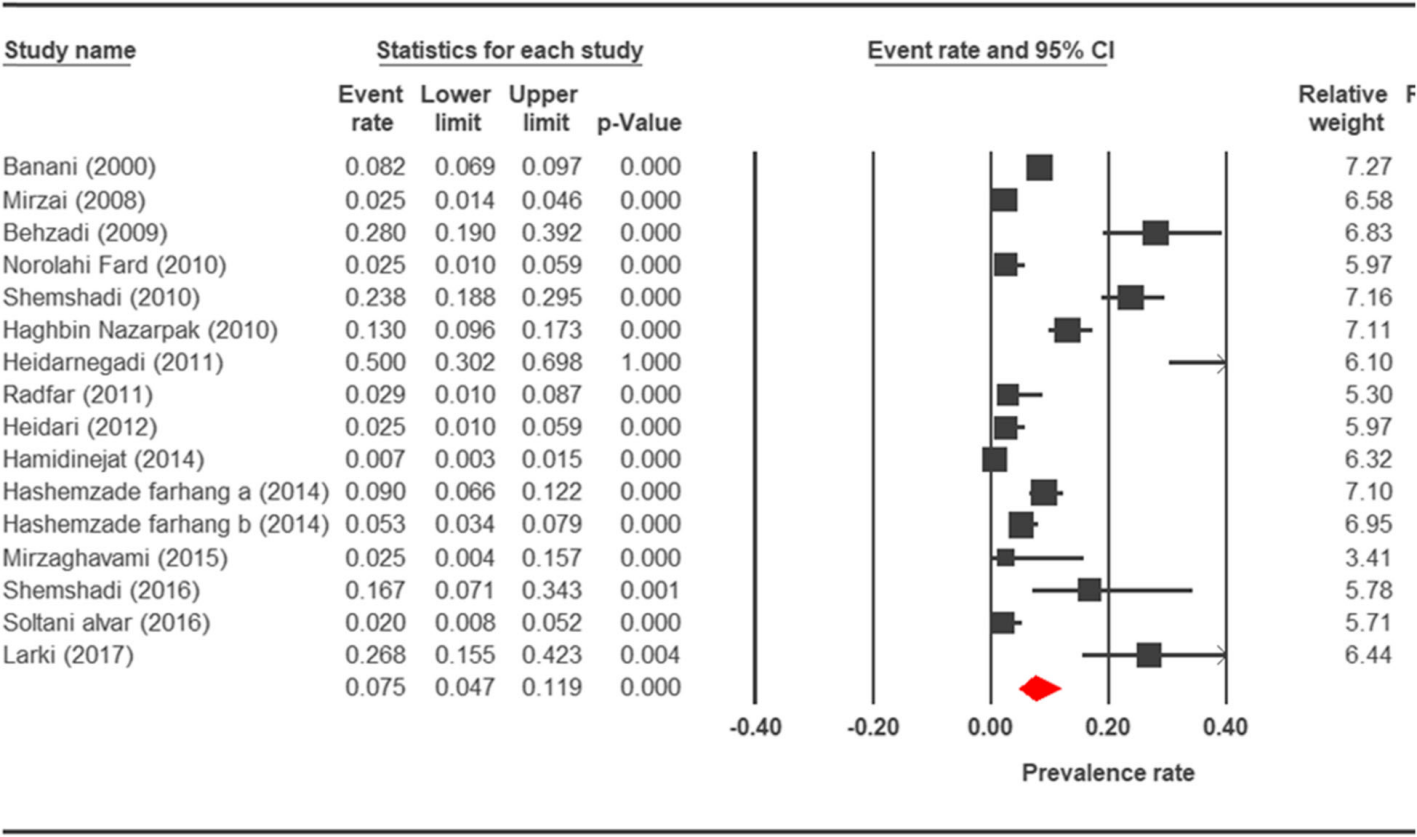
Forest plot diagram showing the prevalence rate of *Cryptosporidium* infection in birds of Iran

**Fig. 3:**
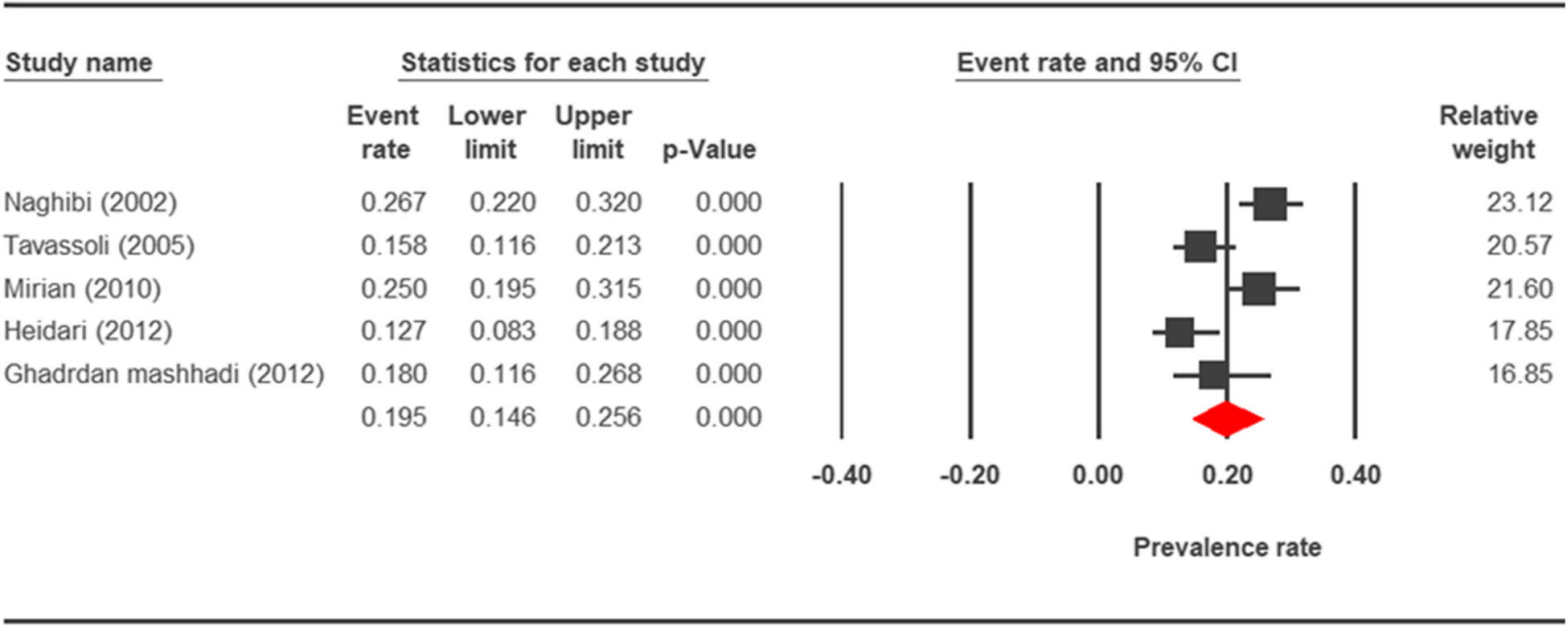
Forest plot diagram showing the prevalence rate of *Cryptosporidium* infection in horses of Iran

**Fig. 4:**
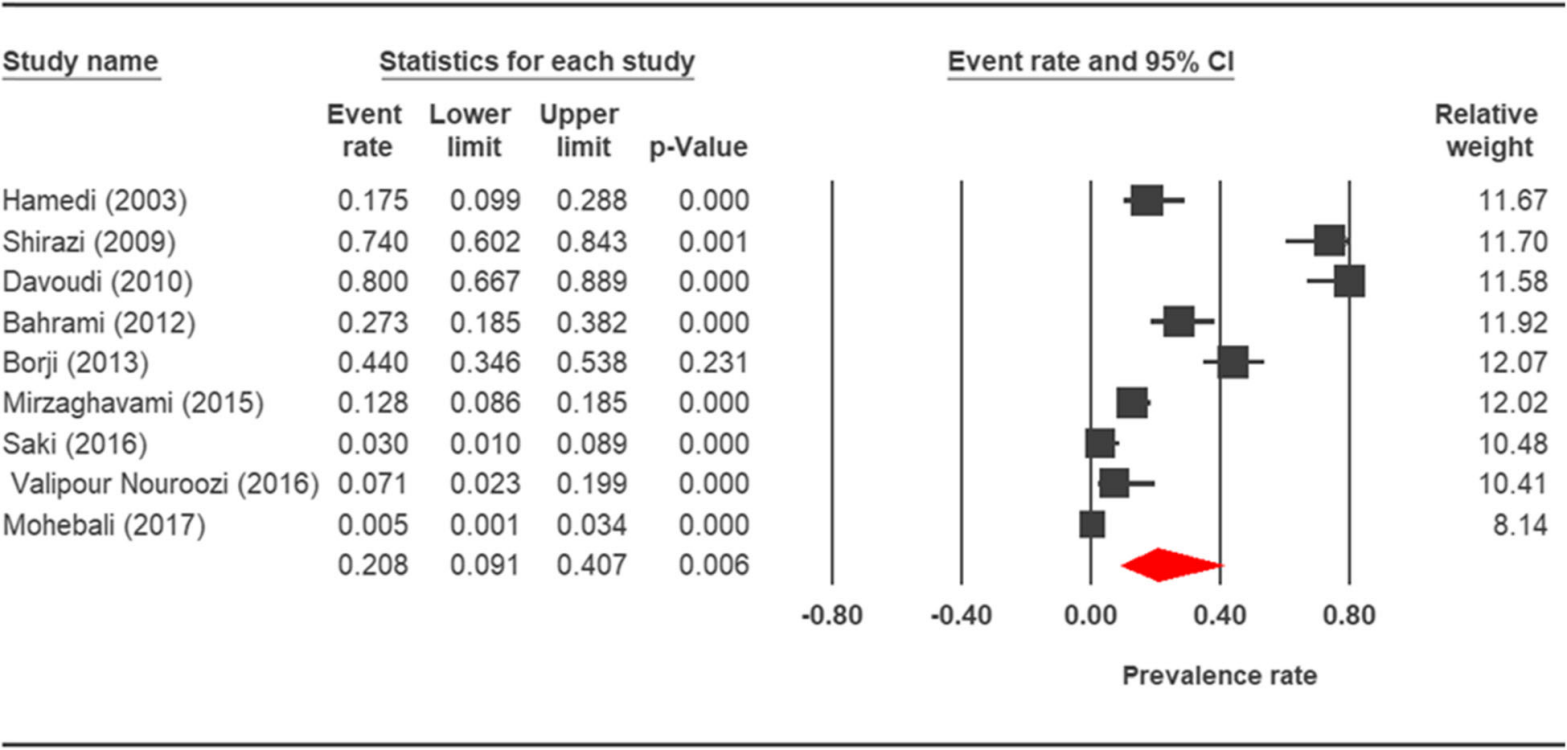
Forest plot diagram showing the prevalence rate of *Cryptosporidium* infection in rodents of Iran

**Fig. 5:**
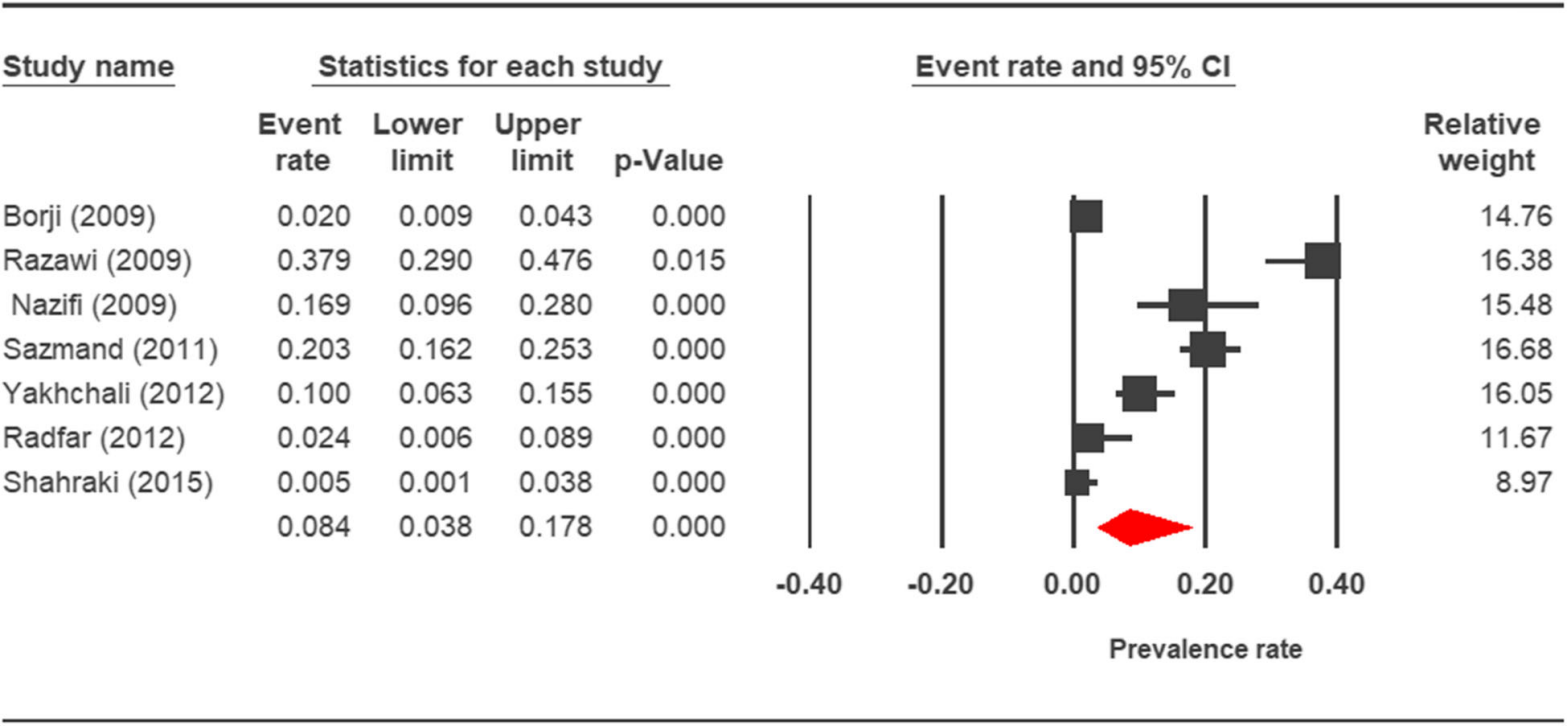
Forest plot diagram showing the prevalence rate of *Cryptosporidium* infection in camels of Iran

**Fig. 6:**
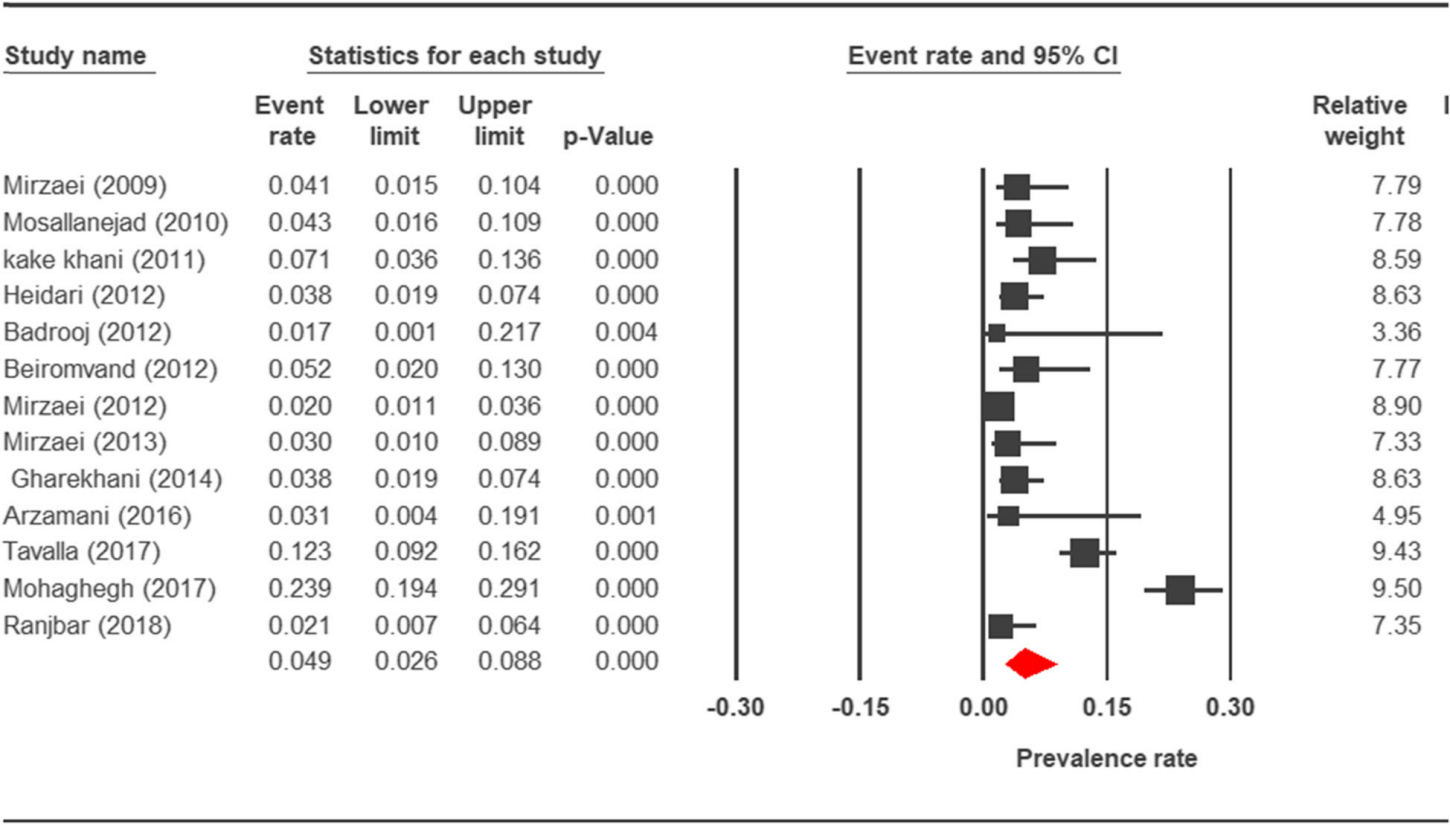
Forest plot diagram showing the prevalence rate of *Cryptosporidium* infection in dogs of Iran

**Fig. 7:**
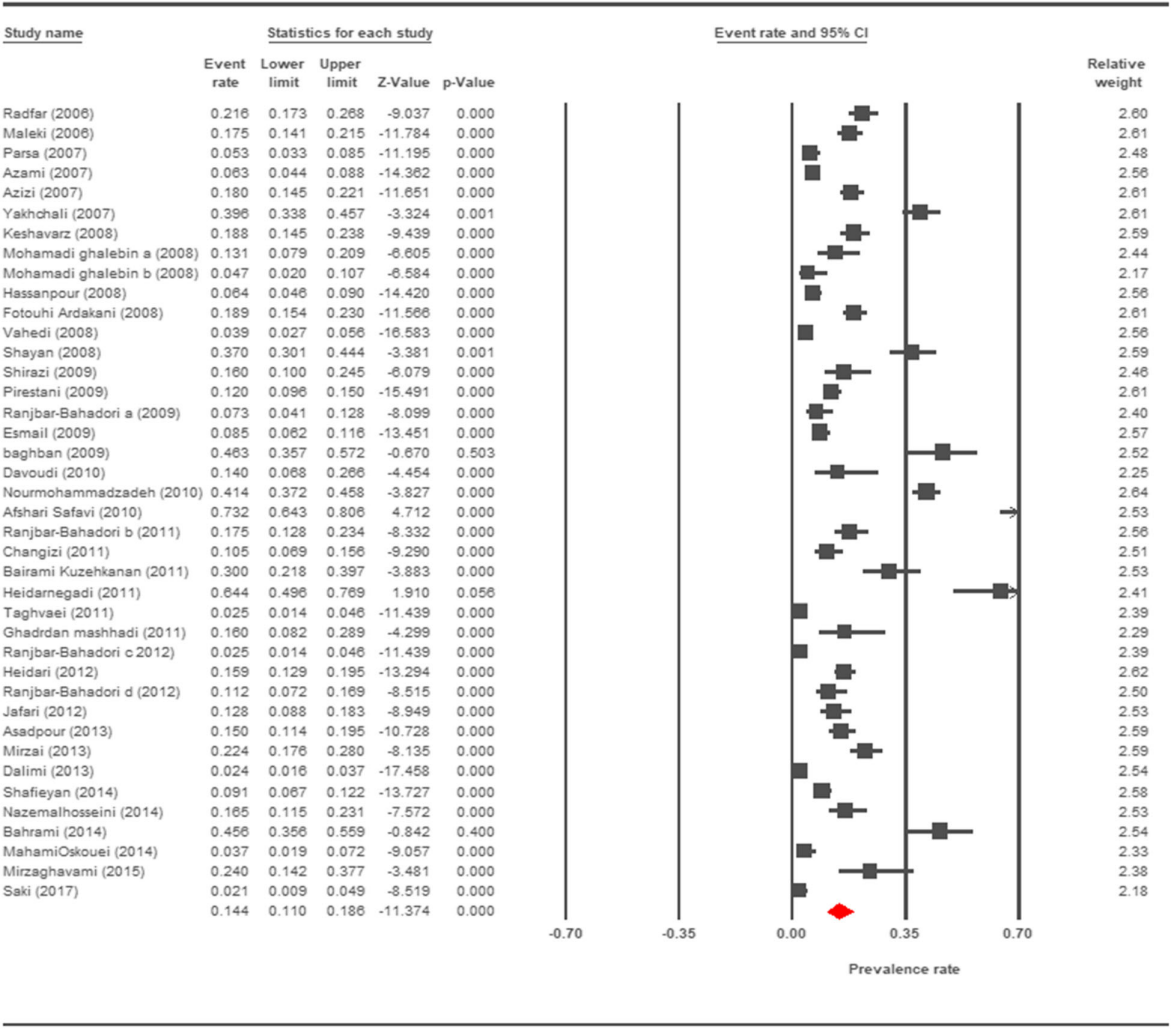
Forest plot diagram showing the prevalence rate of *Cryptosporidium* infection in cattle of Iran

**Fig. 8:**
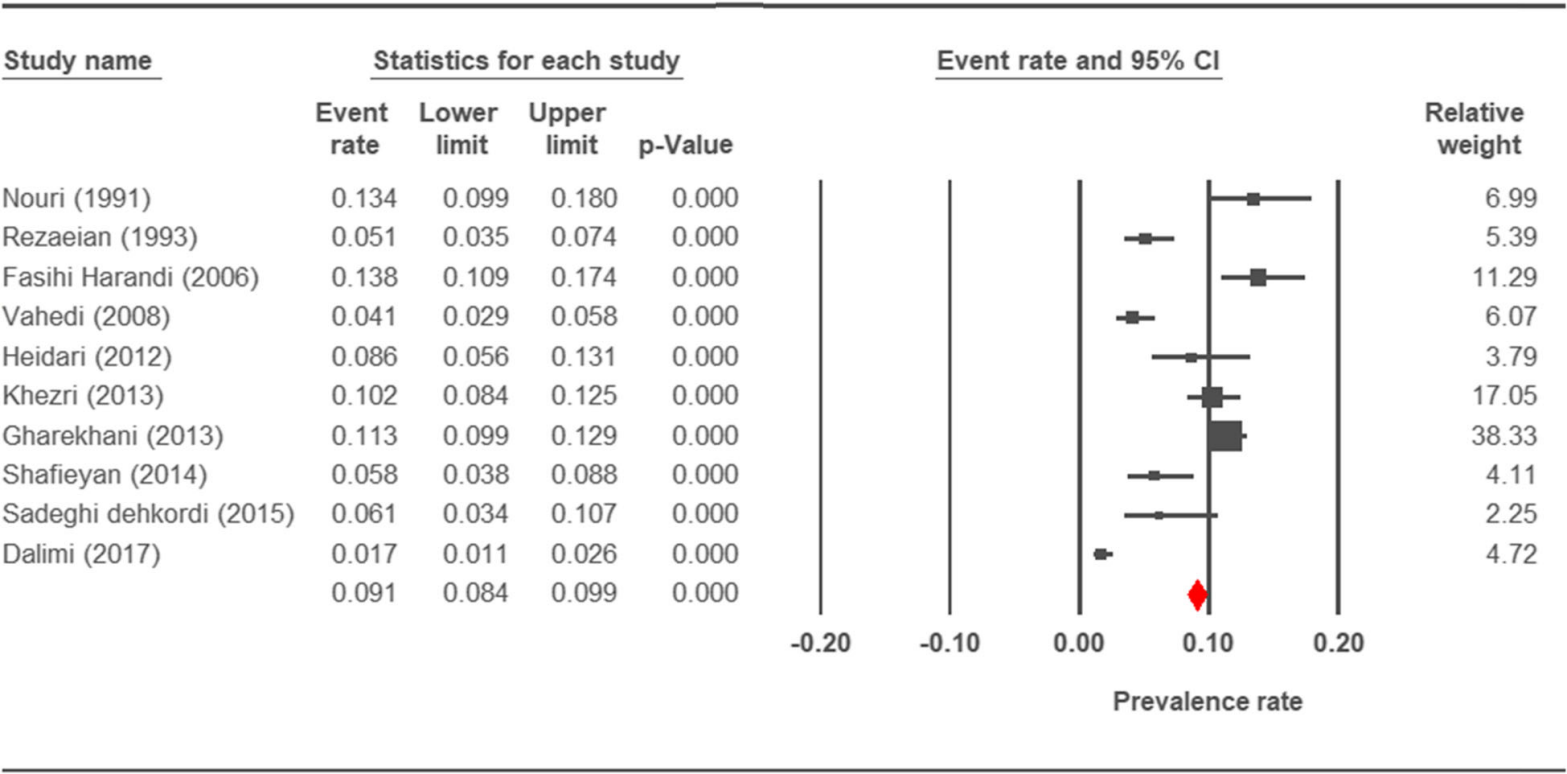
Forest plot diagram showing the prevalence rate of *Cryptosporidium* infection in sheep of Iran

The prevalence rate of cryptosporidiosis within a 27-year period for birds, horses, rodents, camels, dogs, cattle, and sheep in Iran using the random effect model was estimated to be 7.5% (95%, CI = 4.7%, 11.9%), 19.5% (95%, CI = 14.6%, 25.6%), 20.8% (95%, CI = 9.1–40.7%), 8.4% (95%, CI = 3.8%, 17.8%), 4.9% (95%, CI = 2.6%, 8.8%), 14.4% (95%, CI = 11%, 18.6%), and 9.1% (95%, CI = 8.4%, 9.9%), respectively. The forest plot diagrams of the current study are shown in Figs. 2, 3, 4, 5, 6, 7, and 8.

A wide variation was observed in the prevalence estimations among the various studies. The *Q* statistic, df, and *I*^2^ were as follows: 26.63, 15, and 43.74% for birds; 15.15, 8, and 47.18% for rodents; 11.15, 6, and 46.17% for camels; 58.85, 43, and 26.93% for cattle; and 16.64, 10, and 39.93% for sheep, respectively. Low heterogeneity was reported in studies which were conducted on horses as well as the ones on dogs. The statistic factors (*Q* statistic, df, and *I*^2^) were 3.86, 4, 0.00% for horses and 6.07, 12, and 0.00% for dogs.

**Fig. 9:**
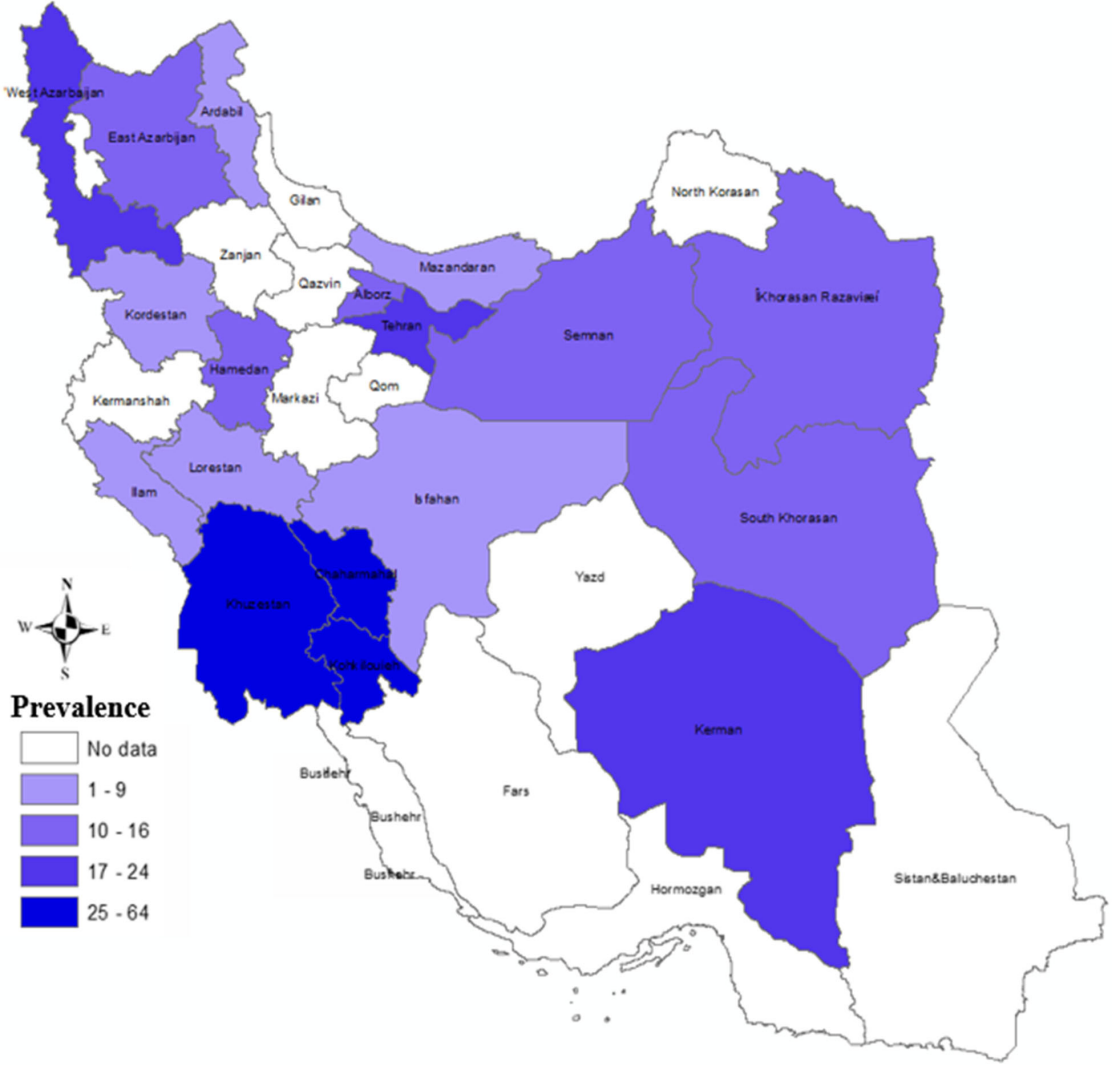
Incidence of Cryptosporidium infection in the Iranian cattle in different provinces [2]

The prevalence rate of *Cryptosporidium* infection in cattle is shown in Fig. 9. The most positive cases of cryptosporidiosis were reported in cattle of West Azerbaijan, Tehran, Khuzestan, Chaharmahal and Bakhtiari, Kohgiluyeh and Boyer-Ahmad, and Kerman provinces. Considerable positivity rates of cryptosporidiosis in cattle were identified in Razavi Khorasan, South Khorasan, Semnan, Hamadan, Alborz, and East Azerbaijan provinces. There were no positive reports of cryptosporidiosis in cattle in other provinces.

## Discussion

Cryptosporidiosis is one of the most important zoonotic diseases which is reported in humans and animals with a worldwide distribution in more than 106 countries and especially in developing countries [91, 92]. To the best of our knowledge, this is the first systematic review and meta-analyses on the prevalence of animal cryptosporidiosis in Iran.

The present study showed that the average prevalence rate of cryptosporidiosis in birds was 7.5% in Iran. Additionally, the prevalence rate of cryptosporidiosis in animals in Ahvaz, southwestern of Iran was reported as 50% [86] while it was shown that in Gilan, north of Iran, this rate was 17% [84]. In a study by Jasim and Marhoon, it has shown that in Iraq, which is a neighboring region of Iran, the cryptosporidiosis prevalence rate in wild and domestic birds was 58.1% [93]. Even though this region is near the southwest of Iran, the prevalence was higher than in Iran. Moreover, a prevalence of 49% was shown in Mexico [94], and in Brazil, 76% of birds were infected by *Cryptosporidium* [95]. Changes in the prevalence seen in various reports indicate that the probability of transmitting the parasite is higher among animals living together on farms and next to each other compared to other studies that have examined individual specimens.

Preventive efforts by Iranian authorities related to awareness of zoonotic diseases, control of stray dogs, and a low population of pet dogs have increased the possibility of transmission of the disease from livestock [96]. On the one hand, the stray dogs are the largest group of dogs in both rural and urban areas in Iran which usually become infected by roaming in human neighborhoods and feeding on contaminated residues. On the other hand, domestic dogs are not restricted to the limited area of houses or farms. Stray dogs are allowed to wander around, so it increases the risk of zoonotic infections in rural habitats. In this study, the overall prevalence of cryptosporidiosis between dogs was found to be 4.9% in Iran. There are various reports of cryptosporidiosis prevalence in the different geographical regions of Iran. Mohaghegh et al. reported a prevalence of 21.7% and 25.4% of cryptosporidiosis respectively in domestic and stray dogs of Kermanshah [61]. Furthermore, the 12.3% prevalence rate of cryptosporidiosis in dogs was observed in Ahvaz [60]. These results are higher than the data which were obtained in other regions of Iran, specifically 5% in Chenaran, northeast of Iran [56]; 7% in Ilam [97]; 2% in Kerman, southeast of Iran [98]; 2.9% in Urmia, northwest of Iran [99]; 2.14% in Isfahan, center of Iran [62]; and 3.8% in Hamadan, west of Iran [17]. The high prevalence of cryptosporidiosis in some areas, for instance, Kermanshah Province, indicated that humans are at serious risk of *Cryptosporidium* infection. Furthermore, the infection can spread vastly and cause severe problems in the community.

Epidemiological studies on cryptosporidiosis infection indicated that the prevalence of *Cryptosporidium* species in dogs is very different in various countries changing from 0 to 52.7%. These differences might be attributed to several factors, such as geographical area, sample size, keeping a dog, correlation with other hosts (such as goat, sheep, horse, cattle, and pig), different species of *Cryptosporidium*, and sampling procedures as well as diagnostic methods [100, 101]. The current results imply that the prevalence rate of cryptosporidiosis in Iran is higher than in countries such as the Czech Republic with 1.4% [102], Thailand with 2.1% [103], Brazil with 2.4% [104], Japan with 3.9% [105], and Spain with 4.1% [106], but lower than in Nigeria with 18.5% [107] and Romania with 52.7% [108].

Rodents could be potential reservoir hosts for zoonotic cryptosporidiosis. During extensive epidemiological studies that have been performed throughout the world, infection in rodents was highly varied from 7.6% in Maryland [109] to 63% in United Kingdom [110]. Other studies showed different statistic in different countries. Specifically, 8.2% in northern Australia [111], 11/5% in China [112], 24.3% in Italy [113], 25.8% in Philippines [114], and 32.8% in the United States of America [115] were reported. The average prevalence rate of cryptosporidiosis in rodents in Iran was estimated as 20.8% in this study. Similar studies in different geographical regions of Iran showed diverse range of prevalence. The frequency of rodent’s cryptosporidiosis in Meshgin shahr, Tehran, Shooshtar, and Ahvaz was 0.5% [75], 27.3 % [71], 7.1% [74], and 3% [1], respectively. In the parasite investigation of rodents of Mashhad, none resulted to be were contaminated (0%) [72].

The average prevalence of cryptosporidiosis in sheep was found as 9.1%. Prevalence of cryptosporidiosis was reported as 1.69%, 5.8%. 6.1% and 8.6% in Tehran [20], Lorestan [18], Sanandaj [19] and Hamadan [15], respectively. Majewska et al found similar results in the west-central region of Poland (10.1%) [116] but lower rates were detected in Australia (24.5%) [117], and China (4.8%) [118].

The prevalence rate of cryptosporidiosis in cattle was 1.5% in Japan [119], 35.7% in Vietnam [120], 20.6% in Turkey [121], 40.6% in Canada [122], and 40.6% in the USA [123].

However, according to this systematic review and meta-analysis, the prevalence of cryptosporidiosis in cattle and calves was 14.4% in Iran, and the prevalence rate in various geographical regions was as follows: 2.1% in Ahvaz [73], 9.07% in Lorestan [18], 16.45% in Qazvin [26], 22.3% in the city of Urmia [48], and 28.3% in the city of Mashhad [47]. Furthermore, our study showed that the prevalence rate of cryptosporidiosis in camels and horses was 8.4% and 19.5%, respectively.

It was suggested in this study that the distribution of *Cryptosporidium* differs among geographical regions. Therefore, the study location might be one of the most determinant factors in cryptosporidiosis distribution. The highest prevalence rate (50%) of cryptosporidiosis was observed in Khuzestan Province [86]. This high prevalence might be attributed to the high temperature and humidity of the southwestern regions of Iran as well as the people’s lifestyle, who have a high level of seafood consumption compared to other regions. Additionally, the immigration of birds to the south of Khuzestan Province may transmit parasite protozoan infection.

## Conclusion

The relatively high prevalence of cryptosporidiosis infection among animals in Iran, mostly among sheep, cattle, and calves, shows the enzootic status of cryptosporidiosis in the investigated areas and may be a threat to the inhabitants. Our data offer important information about the epidemiology of cryptosporidiosis among animals in Iran, which could be useful for managing and controlling programs for the disease. Further investigation and monitoring will be required to expand the surveillance and control policies in order to reduce the prevalence of Cryptosporidiosis among livestock and consequently decrease the economic damages and public health hazards in Iran.

## Supplementary Information

**Additional file 1.** PRISMA Checklist.

## Data Availability

Input data for the analyses are available from the corresponding author on request.

## Abbreviations

HIV: Human immunodeficiency viruses
CI: Confidence interval
